# Developmental and loco-like effects of a swainsonine-induced inhibition of *α*-mannosidase in the honey bee, *Apis mellifera*

**DOI:** 10.7717/peerj.3109

**Published:** 2017-03-16

**Authors:** Laura Wedd, Regan Ashby, Sylvain Foret, Ryszard Maleszka

**Affiliations:** 1 Research School of Biology, Australian National University, Canberra, Australia; 2 Centre for Research in Therapeutic Solutions, Health Research Institute, Faculty of Education, Science, Technology and Mathematics, University of Canberra, Canberra, Australia

**Keywords:** Deleterious epialleles, Metabolism, Mannosidosis, Toxic alkaloid, Epigenetic variation

## Abstract

**Background:**

Deficiencies in lysosomal a-mannosidase (LAM) activity in animals, caused either by mutations or by consuming toxic alkaloids, lead to severe phenotypic and behavioural consequences. Yet, epialleles adversely affecting LAM expression exist in the honey bee population suggesting that they might be beneficial in certain contexts and cannot be eliminated by natural selection.

**Methods:**

We have used a combination of enzymology, molecular biology and metabolomics to characterise the catalytic properties of honey bee LAM (AmLAM) and then used an indolizidine alkaloid swainsonine to inhibit its activity *in vitro* and *in vivo*.

**Results:**

We show that AmLAM is inhibited *in vitro* by swainsonine albeit at slightly higher concentrations than in other animals. Dietary exposure of growing larvae to swainsonine leads to pronounced metabolic changes affecting not only saccharides, but also amino acids, polyols and polyamines. Interestingly, the abundance of two fatty acids implicated in epigenetic regulation is significantly reduced in treated individuals. Additionally, swainsonie causes loco-like symptoms, increased mortality and a subtle decrease in the rate of larval growth resulting in a subsequent developmental delay in pupal metamorphosis.

**Discussion:**

We consider our findings in the context of cellular LAM function, larval development, environmental toxicity and colony-level impacts. The observed developmental heterochrony in swainsonine-treated larvae with lower LAM activity offer a plausible explanation for the existence of epialleles with impaired LAM expression. Individuals carrying such epialleles provide an additional level of epigenetic diversity that could be beneficial for the functioning of a colony whereby more flexibility in timing of adult emergence might be useful for task allocation.

## Introduction

Alleles with undesirable phenotypic effects are subject to the pressure of natural selection that acts by eliminating less fit variants out of a population (Eyre-Walker et al., 2002; Sunyaev et al., 2001). Yet, individuals carrying such alleles exist in human and other populations (Eyre-Walker & Keightley, 2007; Eyre-Walker, Woolfit & Phelps, 2006; Kondrashov, 1995; Trask et al., 2016) posing a question what keeps natural selection from getting rid of them? Recently, we have uncovered several differentially methylated obligatory epialleles of the gene coding for lysosomal α-mannosidase (AmLAM, GB44223) in the honey bee population (Wedd, Kucharski & Maleszka, 2016; Wedd & Maleszka, 2016). These epialleles correlate with context-dependent changes to AmLAM gene expression, with certain epialleles found to be associated with a lower expression of AmLAM. In humans and animals a LAM deficiency leads to a severe impairment in cellular function, which can lead to death (Borgwardt, Lund & Dali, 2014; Malm & Nilssen, 2008). Taking into consideration the severe consequences of LAM deficiency in humans and animals, the persistence of such alleles including those associated with lower expression of LAM in the honey bee population is somewhat puzzling. This study was designed to shed more light on this interesting phenomenon and explores the effects of swainsonine, a natural inhibitor of LAM, on honey bee development.

LAM belongs to family GH38 of the glycoside hydrolases, or class II α-mannosidases that play a critical role in catalysing the hydrolysis of asparagine-linked oligosaccharides during biosynthesis and catabolism across a number of cellular contexts (Daniel, Winchester & Warren, 1994; Gonzalez & Jordan, 2000). These enzymes are classed into a number of different subfamilies based on their localisation, biochemical properties and substrate specificities; they include lysosomal α-mannosidase, likely to have a role in scavenging degraded glycoproteins, the cytosolic α-mannosidase and the well-characterised Golgi α-mannosidase (Henrissat & Bairoch, 1993; Nemcovicova et al., 2013).

LAM has been characterised across a number of eukaryotes. It is localised within the lysosome, typically has a pH optimum of 4.0–4.5, is activated by Zn^2+^, and has a broad substrate specificity, hydrolysing at α1,2, α1,3 and α1,6 linkages during catabolism (Daniel, Winchester & Warren, 1994; Gonzalez & Jordan, 2000; Henrissat & Bairoch, 1993; Nemcovicova et al., 2013). Structural characterisation of this enzyme in *Bos taurus* (cattle) and *Drosophila melanogaster* (vinegar fly) has shown that LAM is proteolytically cleaved into five peptides (A–E), with peptides A and B forming the active-site domain. Mutations disrupting this active site and other critical residues have been well described in cattle, guinea pigs, cats and humans and cause the autosomal recessive disease, α-mannosidosis(Berg et al., 1999). In cases of α-mannosidosis the deficiency of LAM leads to the accumulation of partially degraded mannose-rich oligosaccharides within the lysosome causing lysosomal vacuolation, which eventually leads to mental retardation, hearing loss, skeletal abnormalities and immune deficiency (Borgwardt, Lund & Dali, 2014).

Effects similar to α-mannosidosis have been observed in cases of locoism, a condition which commonly occurs in livestock that consume the common locoweed, so named for its neurological effects. Grazing on the locoweed, which includes several species such as *Swainsona canescens*, *Astragalus mollissimus*, and *Oxytropis lambertii*, induces a phenocopy of α-mannosidosis, which occurs when these plant species contain the indolizidine alkaloid, swainsonine (Molyneux & Panter, 2009). Swainsonine is produced by fungal endophytes that colonise these species, and causes significant livestock losses worldwide (Cook, Gardner & Pfister, 2014). It is believed to bind reversibly to LAM, inhibiting its activity, and when consumed for prolonged periods will induce a phenocopy of α-mannosidosis (Dorling, Huxtable & Colegate, 1980).

This sensitivity of LAM to swainsonine has been documented in a number of species including *D. melanogaster*, sheep and cattle (Nemcovicova et al., 2013). In livestock the onset of symptoms typically occurs after 2–3 weeks of continuous grazing on poisonous plants. Livestock chronically exposed to swainsonine exhibit decreased weight, hyper-excitability, nervousness, muscle incoordination, and with continued exposure will die; where the individual is pregnant abortions and birth defects occur (Molyneux & Panter, 2009). This exposure has been shown to cause the accumulation of oligosaccharides such as Man5GlcNAc2 and Man4GlcNAc2 in the kidney and brain of livestock and the resulting lysosomal vacuolation occurs across most cells, with eventual neuronal vacuolation causing the described neurological effects.

The Western honey bee *A. mellifera* is known to forage on species such as *Astragalus* and *Oxytropis* but only anecdotal evidence indicates that such foraging can lead to deleterious effects. Early reports indicate that the locoweed is toxic to the bee; adult bees have been found dead on *Astragalus lentiginosus* blooms, with additional reports indicating that brood are killed, pupae mummify and die, and queens within affected colonies stop laying (Beckwith, 1898; Vansell & Watkins, 1934). Although the occurrence of colony collapse disorder (CCD) has led to substantial research into the effects of various environmental toxins and pesticides on honey bee health, the sensitivity of the honey bee LAM to swainsonine has not been investigated.

In view of the severe consequences associated with an inactive LAM and its conserved ubiquitous expression and role in core housekeeping processes, glycoprotein catabolism, the identification of differentially methylated LAM epialleles in the honey bee raises the question: what effects, if any, might such epiallelic variation have on LAM function and phenotype more broadly? To understand the existence of these epialleles and explore LAM function in more detail we utilised swainsonine as a surrogate system to generate individuals with impaired LAM function. As a natural inhibitor of LAM, swainsonine treatment provides a convenient tool to investigate how deficiencies of LAM affect both larval development and adult behaviour.

We have conducted a series of *in vitro* and *in vivo* experiments, including a seminal analysis of a swainsonine-induced metabolome to demonstrate that this alkaloid inhibits α-mannosidase activity in the honey bee and leads to considerable metabolic and developmental changes in exposed individuals. We discuss these findings in the context of LAM function, development and colony-level impacts and propose that differentially methylated epialleles generate an additional level of epigenetic diversity that might be advantageous for a self-organising system.

## Materials and Methods

### Biological material

All honey bee specimens came from our Canberra colonies. Larvae were harvested from brood frames taken from the hive and incubated at 35°C, 80% humidity and snap frozen in liquid nitrogen if required. Tissue dissections were carried out in a standard bee Ringer solution (Bicker, 1995). Larvae were grown *in vitro* as described previously (Kucharski et al., 2008). To facilitate the larval-pupal transition and enhance adult emergence, larvae were transferred just before spinning the cacoon to gelatine capsules with small holes at one end. For more details regarding honey bees and specimen preparations see previous publications (Becker et al., 2016; Kucharski, Foret & Maleszka, 2015; Kucharski, Maleszka & Maleszka, 2016; Wedd, Kucharski & Maleszka, 2016).

### Molecular methods

All molecular protocols are described in detail in our previous publications; RNA extractions (Kucharski, Maleszka & Maleszka, 2007; Wojciechowski et al., 2014), qPCR analyses, quality controls (Becker et al., 2016; Kucharski, Maleszka & Maleszka, 2016), primers and conditions for LAM amplification (Wedd, Kucharski & Maleszka, 2016).

### Enzymatic assay

All larval samples were homogenised in a chilled PBS buffer containing 1 mM ZnCl_2_ and cOmplete™ EDTA-free protease inhibitor cocktail (1X; Roche). 250 μL of buffer was added per 100 mg of tissue, the sample homogenised and centrifuged at 14,000× *g* for 15 min at 4°C and the supernatant collected for the activity assay.

10 µL of each extract was incubated with *p-* nitrophenyl- α-D-mannopyranoside in 100 mM acetate buffer (pH 4.6) in a total volume of 50 µL at 37°C. For the K_*i*_ value and IC_50_ estimates the rate of nitrophenol production was determined in the presence of swainsonine (10 inhibitor concentrations ranging from 5–8,000 nM) across six *p*-nitrophenyl- α-D-mannopyranoside substrate concentrations (0.5 mM, 0.75 mM, 1.0 mM, 2.5 mM, 5.0 mM and 7.0 or 8.0 mM); the K_*i*_ value and IC_50_ range were calculated using the GraphPad Prism curve fitting software. For the standard assay LAM activity was determined with 1.0 mM *p*-nitrophenyl- α-D-mannopyranoside. All reactions were terminated with 250 µL of 100 mM sodium carbonate and *p*-nitrophenyl production measured at 410 nm using a spectrophotometer (Multiskan RC).

LAM activity was determined using a nitrophenol standard curve (1,000 μM, 500 µM, 250 µM, 125 μM, 62.5 µM, 31.25 µM, 15.63 µM and 7.8 µM concentrations), using the following equation:

\begin{eqnarray*}\mathrm{LAM~ activity~ (U/ mL)}& =& \frac{ \left( \mathrm{ODsample}-\mathrm{ODblank} \right) \times \mathrm{total} \mathrm{reaction} \mathrm{vol} \left( \mathrm{mL} \right) }{\mathrm{time}\times  \mathrm{slope}\times \mathrm{sample} \mathrm{vol} \left( \mathrm{mL} \right) } \nonumber\\\displaystyle & & \times ~\mathrm{Dilution} \mathrm{Factor} \left( \mathrm{DF} \right) \end{eqnarray*}

Units: 1 Unit (U) of LAM will catalyse the conversion of 1 µmole of *p-* nitrophenyl- α-D-mannopyranoside to 4-nitrophenol and α-D-mannose per minute at 37°C at the indicated pH (pH 4.6). OD = optical density.

### Metabolomic analyses

Single larvae were hand-homogenised with a plastic pestle for 30 s in 100 µL of chilled (4°C) methanol. A further 400 µL of chilled (4°C) methanol was added to bring the final volume to 500 µL. The samples were sonicated in a sonic bath for 1 min before 40 µL of 0.1mg/mL L-norvaline (MW 117.15g/mol, Sigma N-7627) was added to each sample as an internal standard. Following incubation at 60°C in a water bath for 15 min, with vortexing every 5 min, 280 µL of chloroform was added and mix thoroughly by inversion before incubation in a water bath at 37°C for a further 5 min. After adding 550 µL of water and thorough mixing, the samples were centrifuged at 14,000 rpm for 5 min at room temperature. Aliquots of 50 µL were collected from the upper methanol phase (approx. 500 µL) and placed in brown GC-MS glass vials for analysis. Samples were dried down in a speedivac at 40°C for 1 h before being sent for analysis.

Samples were derivatised in methoxyamine, HCl in anhydrous pyridine (10 uL–20 mg/ml) for 90 min at 37°C, while agitated at 37 rpm, and N-methyl-N-(trimethylsilyl)-trifluoroacetamide (15 uL derivatisation grade, Sigma-Aldrich 394866-10 X 1ML) for 30 min at 37°C, while agitated at 37 rpm, using an online Gerstel Multipurpose Sampler MPS (GERSTEL GmbH and Co.KG, Germany). The derivatised samples were analysed by GC-MS with a full MS scan range of 40–600 m/z, with an acquisition rate of 4 scans/sec, and the solvent delay of 5.6 min (Agilent 7890A, Agilent 5975C MS inert XL EI/CI MSD with triple axis detector, Santa Clara, CA). Samples were injected at a volume of 1uL, with a splitless injector temperature of 230C, and separated on an Agilent J&W VF-5ms 5% phenyl-methyl column (30 m length × 0.25 mm internal diameter, 0.25 um film thickness). The temperature programme applied for GC separation was: 70°C for 1 min, ramping at 15°C/min to 325°C and held for 3 min. Total run time was 21 min with a helium carrier flow rate of 1 ml/min at 12.445 psi. Temperature of the MS transfer line was 250C.

Metabolites were screened using MetabolomeExpress (Carroll, Badger & Harvey Millar, 2010) and AMDIS software package (Halket et al., 1999) and identified using retention indices (C12-C36 n-Alkanes, 5 uL at 147 mg/L) against the Golm Metabolome Database (GMD) (Kopka et al., 2005). The peak areas of detected analytes were normalised against the internal standard L-Norvaline. All readings were measured in triplicate.

## Results

### Swainsonine inhibits mannosidase activity in *A. mellifera*

**Figure 1:**
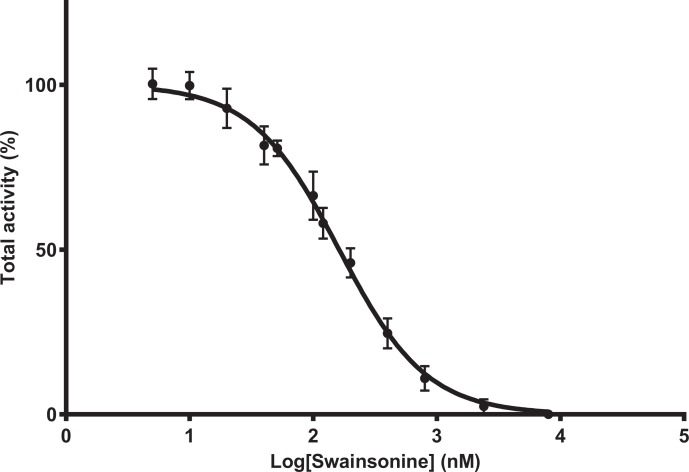
Swainsonine inhibition of mannosidase activity in *A. mellifera*. Tissue extracts were prepared from 48–96 h larvae; individuals from three separate hives were pooled (*n* = 6–10/pool, total of 8 pooled samples) and mannosidase activity determined in the presence of 5–8,000 nM of swainsonine. The percent total activity was calculated as the amount of nitrophenol produced in the presence of swainsonine over the nitrophenol produced in the absence of swainsonine (assay conditions: 1 mM substrate, pH 4.6, 37°C). The IC_50_ is estimated between 145.9 and 179.9 nM, with the average IC_50_ 162 nM (*R*2 = 0.9854, Hill Slope = −1.143).

To determine whether *A. mellifera* is sensitive to swainsonine, mannosidase activity was determined in the presence of swainsonine at concentrations ranging from 5 to 8,000 nM, with the inhibitor and substrate added simultaneously. Maximal inhibition is reached at swainsonine concentrations of 8,000 nM, and 50% of mannosidase activity is inhibited at swainsonine concentrations between 145.9 and 179.9 nM (mean IC_50_ = 162 nM, *n* = 8) (Fig. 1).

### Swainsonine binds in a competitive and reversible manner

To gain insight into the mechanism of action of swainsonine in *A. mellifera* the inhibition constant, K_*i*_, was determined over 10 inhibitor and 6 substrate concentrations. The observed inhibition of mannosidase activity is competitive, with a K_*i*_ value determined over two replicates as 152.3 ± 17.49 nM (95% CI [117.3–187.2]) and 223.6 ± 27.41 nM, (95% CI [168.8–278.4]); the K_*i*_ for swainsonine is therefore estimated to be in range of 135–251 nM (Figs. S1A–S1C). To determine whether swainsonine binding is reversible, extracts were preincubated with a swainsonine concentration near to 10 ×  *K*_*i*_ (∼1,600 nM) followed by a 1:30 dilution into substrate; it was found that little activity was recovered in the preincubated sample after dilution (Fig. S2), which is typical of irreversible binding or reversible inhibitors that dissociate slowly from the enzyme. A further analysis at 5 ×  K_*i*_ (∼800 nM) followed by 1:5 dilution into substrate showed a slight recovery of activity after 60 min, which suggests that binding is reversible (Fig. S2B).

### The effects of swainsonine exposure on mannosidase activity and AmLAM gene expression in larvae

To ascertain the effects of swainsonine on honey bee development, larvae were reared in the laboratory on a larval diet containing 20 µM and 100 µM concentrations of swainsonine for a period of eight days, prior to their transfer into capsules for pupation.

A larval diet containing swainsonine did not increase mortality within the first eight days of development; 20% (*N* = 10∕50) of larvae reared in the control condition did not survive, similar to those reared on a diet containing 20 µM (21%, *N* = 14∕66) and 100 µM (10.5%, *N* = 7∕67) of swainsonine, and there was no significant difference in the number of larvae that died between conditions (*F*(2, 18) = 0.29, *p* = .75). Additionally, no well-defined phenotypic differences occurred and no significant differences in weight were observed at day 4 (*F*(2, 33) = 0.52), *p* = .60, by one-way ANOVA), or day 8 (*F*(2, 33) = 2.92, *p* = 0.07, by one-way ANOVA) between conditions.

**Figure 2:**
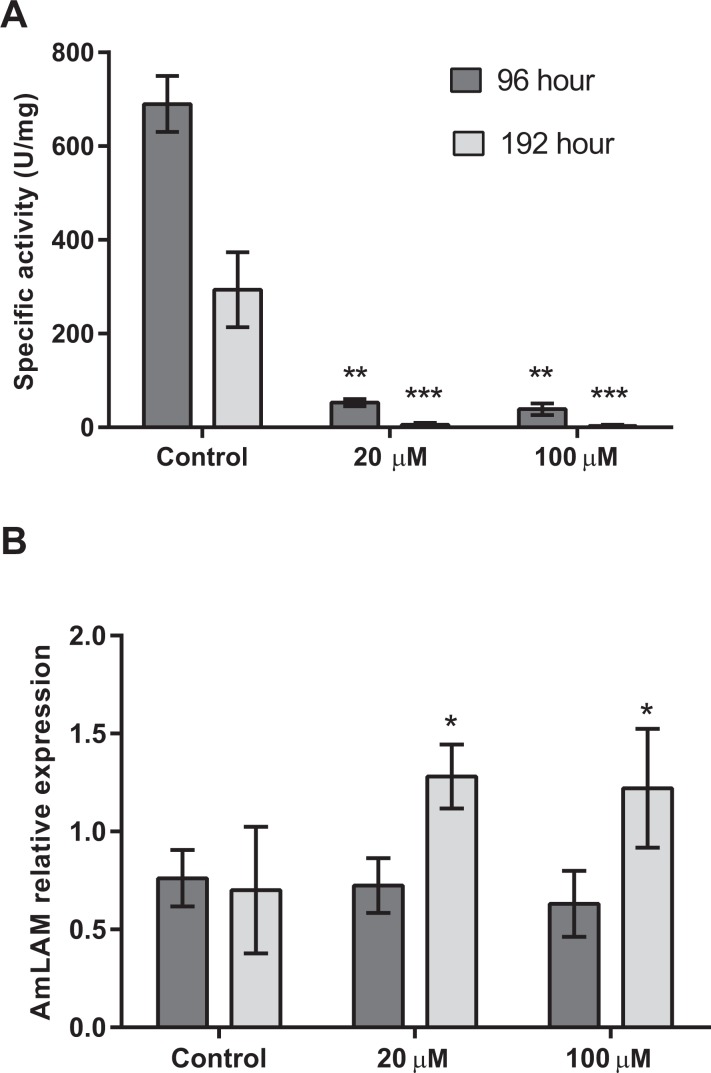
Mannosidase activity and AmLAM gene expression in larvae reared on a diet containing 20 µM and 100 µM of swainsonine. Larvae were reared in the laboratory on a larval diet containing 20 µM and 100 µM of swainsonine, and mannosidase activity (A) and AmLAM gene expression (B) determined in whole individuals at 96 h (4 day exposure) and 192 h (8 days exposure). A significantly lower level of mannosidase activity was observed in treated individuals, with an increase in AmLAM gene expression observed after a prolonged exposure of 8 days. The level of significance is indicated by ^∗^*p* < .05; ^∗∗^*p* < .01; ^∗∗∗^*p* < .001.

Four days after exposure to swainsonine, (96 h larval stage, Fig. 2) a significant decrease in mannosidase activity occurs; a 13-fold and 18-fold higher level of mannosidase activity was observed in larvae fed the control diet (*M* = 690 ± 60 U/mg) than in the 20 µM (*M* = 53 ± 8 U/mg, *t*(3) = 3.18, *p* = .0002; *d* = 14.90) and 100 µM (*M* = 39 ± 12 U/mg, *t*(3) = 3.18, *p* = .0002; *d* = 15.05) conditions, respectively, Fig. 2A. No significant effect of swainsonine treatment on the level of AmLAM gene expression was observed between groups (*F*(2, 12) = 0.98), *p* = .40, by one-way ANOVA (Fig. 2B) suggesting that transcriptional profiling is not sufficient to evaluate the extent to which this alkaloid affects its expression after exposure for only four days.

Further exposure to swainsonine increases mannosidase inhibition. Eight days after exposure to swainsonine (192 h larval stage) a 54-fold and 95-fold higher level of mannosidase activity was observed in the control group (*M* = 294 ± 80 U/mg) than in the 20 µM (*M* = 6 ± 4 U/mg, *t*(3) = 3.18, *p* = .006; *d* = 5.09) and 100 µM (*M* = 3 ± 2 U/mg, *t*(3) = 2.45, *p* = .005; *d* = 5.13) conditions, respectively, Fig. 2A. Additionally, whilst no effect on AmLAM expression was observed at day 4, a significantly higher level of AmLAM expression was observed in larvae fed a diet containing 20 µM of swainsonine (*M* = 1.28 ± 0.03, *t*(6) = 2.45, *p* = .01; *d* = 7.62), and 100 µM of swainsonine (*M* = 1.22 ± 0.09, *t*(8) = 2.31, *p* = .03; *d* = 5.30) than in larvae reared on a control diet (*M* = 0.7 ± 0.1), Fig. 2B.

After day 8 larvae were transferred to gelatine capsules for pupation and emergence, which typically occurs at day 21–22. Of the control group 60% (*N* = 6∕10) survived pupation and all surviving individuals emerged by day 22. Of the larvae reared on a diet containing 20 µM of swainsonine 45.5% survived (*N* = 5∕11) and by day 22 only 60% (*N* = 3) of surviving individuals emerged. For those larvae reared on a diet containing 100 µM of swainsonine 73% survived pupation (*N* = 14∕19) and by day 22 only 57% (*N* = 8∕14) of surviving individuals emerged.

### Swainsonine exposure causes a developmental delay and loco-like symptoms

The aforementioned findings suggest that swainsonine may cause a delay in development, with fewer treated individuals (∼60%) emerging by day 22. To confirm these findings the developmental time course and survival of individuals reared on a diet containing 100 µM of swainsonine was assessed.

Within the first eight to nine days of exposure to swainsonine no increase in mortality was observed. 19.8% mortality occurred in the control condition (*N* = 19∕96) and 20.2% mortality occurred in larvae reared on a diet containing 100 µM of swainsonine (*N* = 18∕89) by day 8. On day 9 the mortality of swainsonine treated individuals increased to 30.4% (*N* = 27∕89), but was not found to be significantly higher than the mortality rate of the control condition (20.8%, *N* = 20∕96; *Z* =  − 1.48, *p* = .14).

Typically laboratory reared larvae are large enough for transfers into capsules by day 8–9. Of those surviving larvae fewer swainsonine treated individuals (24%; *N* = 16∕67) were ready for pupation than in the control group on day 8, where a significantly higher proportion of individuals were ready for pupation (46%; *N* = 34∕74; *Z* = 2.74, *p* = 0.006), suggesting that swainsonine may decrease the rate of larval growth. However, by day 9 all remaining larvae across both conditions were ready for pupation, with the exception of a small percent of individuals that failed to thrive (1.3%, *N* = 1∕74, of control and 3.0%, *N* = 2∕67, of swainsonine treated larvae).

### Effects of swainsonine during pupal development

**Figure 3:**
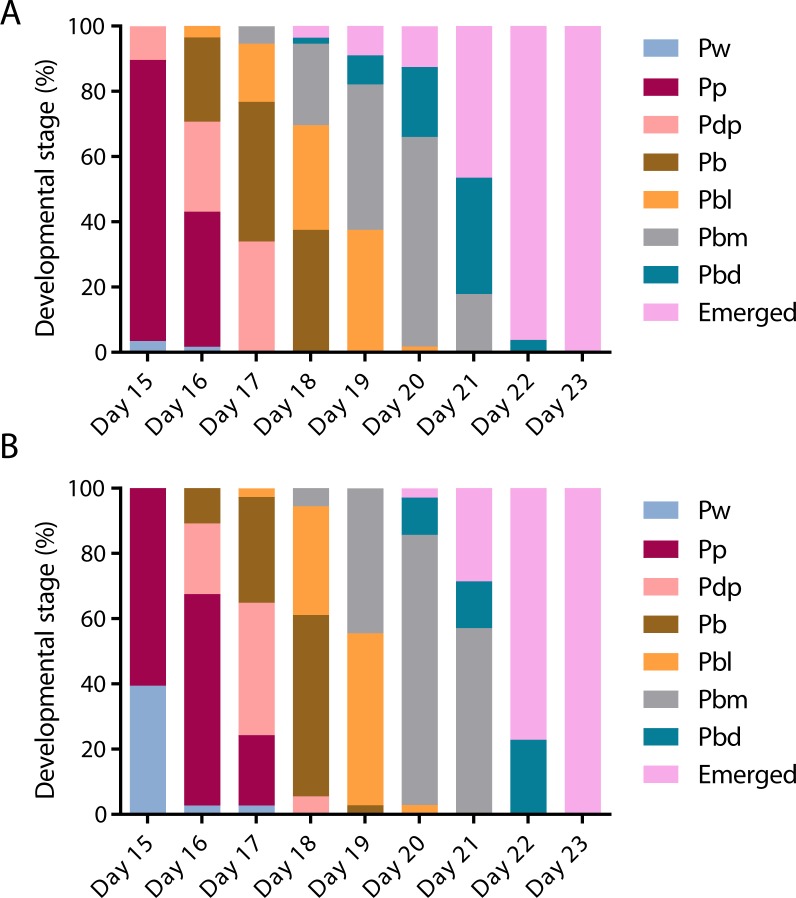
The timing of pupal developmental phases in (A) untreated and (B) swainsonine treated individuals. The colouration of the compound eye and thorax was monitored to assess the rate of development. During early pupation both the eye and body remain white (Pw). Colouration begins with the eyes, which transition from white to pink (Pp), to dark pink (Pdp) and finally to brown (Pb). Once the eyes darken to brown the thorax begins to develop colour, and the body transitions from light in colour (Pbl), to medium (Pbm) and finally dark (Pbd).

To assess the effect of swainsonine on pupal development individual pupal phases, as determined by the colouration of the compound eye and thorax (Rembold, Kremer & Ulrich, 1980) were assessed from day 15 until day 23, when all surviving individuals had emerged (Fig. 3).

On day 15 there was a significantly higher proportion of surviving individuals in the control group (*N* = 50∕58) that had reached the Pp (pink eye, white thorax) phase than the swainsonine treated group (*N* = 23∕38; *Z* = 2.88, *p* = .004). Additionally, on day 15 a significantly higher proportion of swainsonine treated individuals remained in the earlier Pw (white eye, white thorax; *N* = 15∕38) developmental phase than the control group (*N* = 2∕58; *Z* =  − 4.52, *p* < .00001).

Significant effects were seen again on day 17, where a higher proportion of the control group had entered the Pbl (brown eyes, light pigmentation of the thorax) developmental phase (*N* = 10∕56) than the swainsonine treated group (*N* = 1∕37, *Z* = 2.22, *p* = .03). At day 21 a significantly higher proportion of the swainsonine treated group (*N* = 18∕35) remained in the Pbm (brown eyes, medium pigmentation of the thorax) phase than the control condition; on day 21 a higher proportion of control individuals were in the Pbd (brown eyes, dark pigmentation of the thorax) phase or had emerged (*N* = 10∕56; *Z* =  − 3.34, *p* = .0007).

At no time point was there a greater proportion of swainsonine treated individuals in a more advanced developmental stage than the control group, and the onset of each developmental phase was seen to be delayed by approximately one day in treated individuals. During pupation although a higher mortality rate was observed in treated individuals (37.0%, *N* = 20∕54) than the control condition (23.2%, *N* = 16∕69) this difference was not significant (*Z* =  − 1.68, *p* = 0.093), suggesting that swainsonine treatment only marginally affects survival during pupation.

### Swainsonine treatment during development increases mortality

Given that swainsonine was not found to increase mortality during larval stages, but appeared to have marginal effects on survival during pupation we tested the overall effect of swainsonine on survival into adulthood. Mortality in swainsonine treated individuals (55.1%, *N* = 49∕89) across all developmental stages, from early larval to emergence, was significantly higher than control individuals (38.5%, *N* = 37∕96; *Z* = 2.25, *p* = .02), indicating that swainsonine exposure during development reduces the likelihood of an individual surviving into adulthood.

### Effects of swainsonine treatment on adult emergence and post emergence survival

**Figure 4:**
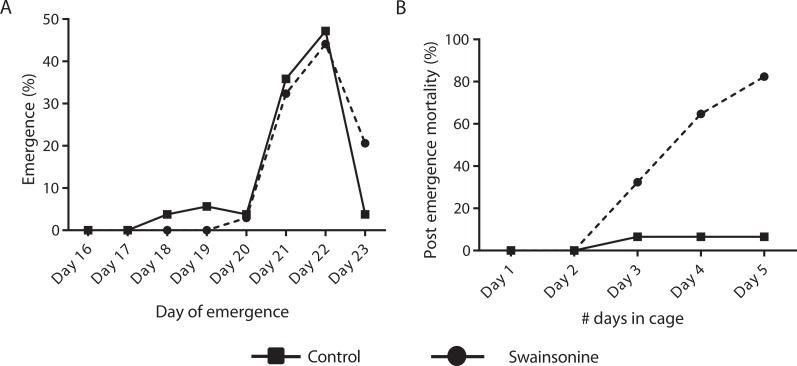
The timing of emergence and survival of newly emerged bees exposed to swainsonine. (A) The percentage of individuals emerging between days 16–23; significant differences were observed only at day 23. (B) The percentage of caged newly emerged bees that did not survive over a five day period after emergence (day 1 in cage = day 21 of emergence).

Of the individuals transferred for pupation 76.8% of the control condition (*N* = 53∕69) and 63.0% (*N* = 34∕54) of larvae exposed to swainsonine emerged successfully. All surviving individuals had emerged by day 23 and no significant difference in the emergence rate was observed between conditions (*Z* = 1.68, *p* = 0.093). Whilst a higher percentage of control individuals (13.3%, *N* = 7∕53) emerged early, between days 18–20, this was not significantly higher than for swainsonine treated individuals (3.0%, *N* = 1∕34; *Z* = 1.62, *p* = .11). The majority of individuals in both conditions emerged on days 21 and 22; no significant difference in the proportion of individuals that emerged on day 21 (control = 19/53, swainsonine = 11/34; *Z* = 0.34, *p* = .74) or on day 22 (control = 25/53, swainsonine = 15/34; *Z* = 0.28, *p* = .78) was observed. However, on day 23, a significantly higher proportion of control individuals (96.2%, *N* = 51∕53) had emerged than swainsonine treated individuals (77.1%, *N* = 27∕34; *Z* =  − 2.51, *p* = 0.01), suggesting that swainsonine may marginally delay emergence in some individuals (Fig. 4A).

**Figure 5:**
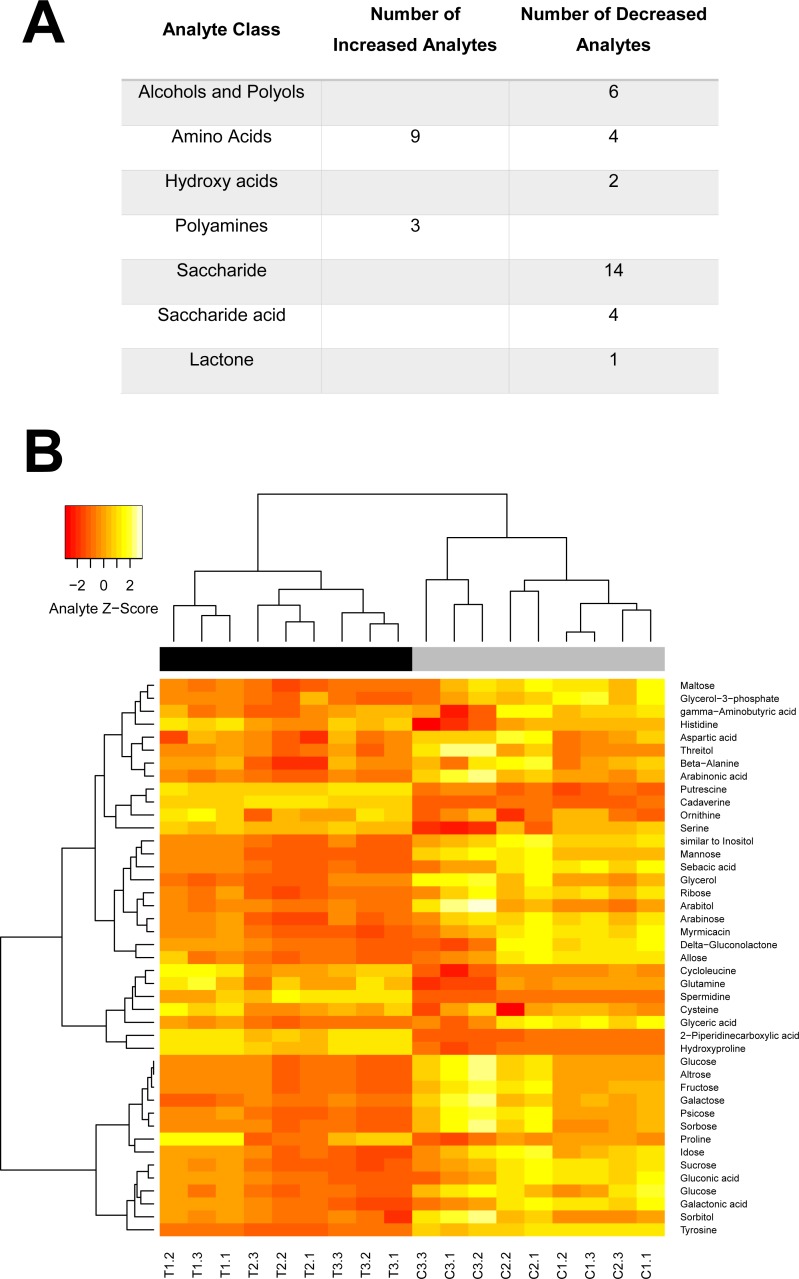
Swainosonine affects distinct metabolic pathways in the honey bee. (A) The number of analytes, per class, that were observed to be elevated or reduced in swainsonine treated larvae relative to age-matched untreated larvae. (B) Heatmap and hierarchical clustering of metabolites that show a statistically significant difference in the levels seen between swainsonine treated larvae and age-matched untreated larvae. Key: C, untreated control sample, T, swainsonine treated samples. The first digit in each name represents the biological replicate number (e.g., C1, C2 and C3), while the second digit after the decimal space indicates the technical replicate number (e.g., C1.1, C1.2 and C1.3). See Table S1 for the full list of metabolites and Fig. S3 for principal component analysis of the GC/MS data for the swainsonine-treated and control samples.

Newly emerged bees were transferred to a cage and mortality and phenotype observed over 5 days. Significant losses for those bees reared on a diet containing swainsonine occurred; by day 5 82.4% (*N* = 28∕34) of treated bees had died, significantly higher than in the control condition where at day 5 only 6.52% of bees had not survived (*N* = 3∕46; *Z* = 6.88, *p* < .00001, Fig. 4B). It was observed that the movement of treated bees was impaired; the honey bees were observed trembling, and appeared to spend more time at the bottom of the cage, suggesting an impairment of locomotor activity.

### Metabolic changes induced by dietary exposure to swainsonine

Given the critical role of α-mannosidases in the maturation and degradation of glycoprotein-linked oligosaccharides and previous results suggesting that swainsonie affects various aspects of metabolism, we have analysed the metabolomes of both control and treated larvae using mass spectrometry. As shown in Fig. 5, several clearly-defined categories of metabolites were affected by dietary exposure to this alkaloid. Distinct changes in the metabolism of amino acids, polyamines, saccharides, hydroxyl acids and polyols were observed in swainsonine treated bees. A number of components of the urea cycle, critical for disposing of excess nitrogen, were found to be elevated, including the amino acids aspartate and ornithine. Besides its role in the urea cycle, ornithine serves a number of other roles including as a precursor for the synthesis of the polyamines putrescine, cadaverine and spermidine, all of which showed elevated levels in swainsonine treated bees. Polyamines have been shown to interact with a number of negatively-charged acid residues associated with nucleic acids, phospholipids as well as acid-containing proteins and polysaccharides. Although the role of polyamines has not been fully elucidated, based on their interactions with acid groups, it has been suggested that they are involved in modulating cell growth, ionic channels, protein synthesis, protein kinases, and cell proliferation/death (Michael, 2016). Their elevated levels in swainsonine treated bees, which show reduced rates of developmental growth, may suggest that increased levels of apoptosis are occurring. Swainsonine treated bees also show a significant reduction in the abundance of 19 different saccharides, as well as 7 sugar alcohols, indicative of reduced metabolic output. What is unclear is whether swainsoine directly alters the biochemical pathways underlying saccharide metabolism, or whether changes in saccharide levels are simply the result of a decline in metabolic demand associated with stunted growth.

Interestingly, a number of metabolites unique to fruit, honey (gluconic acid and δ-gluconolactone) or royal jelly (altrose) were found to be altered, suggesting changes in the metabolism of specific dietary compounds in response to swainsonine treatment. There was also a significant reduction in the levels of a number of critical polyols, although the significance of these changes is unclear. These compounds are often produced as carbon storage materials (Diano et al., 2006) and could be utilised by developing honey bees during metamorphosis when no food intake is occurring. Further, the abundance of two fatty acids (myrmicacin and sebacic acid) implicated in epigenetic regulation is significantly reduced in treated individuals. These histone deacetylase inhibitors play a crucial role in honey bees by linking nutritional inputs with global gene regulation (Makino et al., 2016; Maleszka, 2014).

## Discussion

### The mechanism of swainsonine inhibition of mannosidase activity in the honey bee

We provide seminal evidence that *A. mellifera* mannosidase activity is sensitive to the indolizidine alkaloid swainsonine, although the K_*i*_ estimate for *A. mellifera* (Ki: 135–251 nM) is higher than that reported for the mammalian enzyme (Ki: 70 nM), suggesting that *A. mellifera* may be less sensitive to swainsonine than other organisms (Dorling, Huxtable & Colegate, 1980). In accord with similar studies in other system, *in vitro* inhibition indicates that swainsonine is a competitive inhibitor of *A. mellifera* mannosidase activity. Swainsonine binds tightly to the jack bean α-D-mannosidase and this binding is also influenced by preincubation (Kang & Elbein, 1983), and in the case of mammalian lysosomal α-mannosidase swainsonine inhibition is reversible upon dilution (Dorling, Huxtable & Colegate, 1980). Importantly, the mechanism of swainsonine binding is complex and not fully understood. The degree of reversibility is affected by the extent of preincubation, with suggestion that two modes of binding exist, a rapid and irreversible binding, and a slow and reversible mode of binding (Tulsiani, Broquist & Touster, 1985). Initially, we found no recovery of mannosidase activity after preincubation with high (10 × K _*i*−_) swainsonine concentrations, which is typical of irreversible inhibitors. Yet, subsequent analysis at lower (5 × K_*i*_) swainsonine concentrations showed some recovery. This suggests that swainsonine likely inhibits in a reversible manner, but we recommended further mechanism of actions studies to confirm our findings.

Previous studies have also shown that swainsonine inhibits both the lysosomal α-mannosidase and Golgi mannosidase II *in vitro* (Kang & Elbein, 1983), but is believed to primarily inhibit the lysosomal form *in vivo* since swainsonine is more likely to accumulate within the lysosome (Dorling, Huxtable & Colegate, 1980). Since there are three types of α-D-mannosidases in mammals with their pH range from 4.0 to 6.5 in three different subcellular locations (Winchester, 1984), the results presented here are reflective of its effects on the lysosomal enzyme, since the conditions utilised are optimal for the lysosomal form, with assay conditions performed at pH 4.6. This low pH would limit the activity of the Golgi mannosidase II, and in *D. melanogaster* the Golgi mannosidase II enzyme has been shown to have little activity below pH 5.0 (Nemcovicova et al., 2013). Indeed, the IC_50_ inhibition studies indicate the detection of a single enzyme (Hill slope = −1.143), suggesting little interference from the Golgi mannosidase II enzyme. The future purification of the *A. mellifera* lysosomal α-mannosidase and subsequent inhibition and mechanism of action studies will confirm our findings, and provide definitive IC_50_ and K_*i*_ estimates for the honey bee.

### Effects of swainsonine on honey bee development

Our results indicate that a prolonged exposure to swainsonine has sub-lethal effects on larval and pupal development, with newly emerged bees eventually succumbing to the toxic effects of this alkaloid. In early larval stages mannosidase activity is significantly decreased in treated individuals and continued exposure, up until pupation, further decreases activity. This indicates that during larval development exposure to swainsonine via the diet will result in an accumulation of the toxin, and the increase in AmLAM gene expression after eight days of exposure suggests that, *in vivo*, swainsonine does indeed affect the lysosomal α-mannosidase, as has been reported in other organisms. Such an increase in gene expression may indicate a compensatory mechanism against LAM inhibition.

Exposure to swainsonine causes a subtle decrease in the rate of larval growth, and this developmental delay continues throughout pupation, where the development of swainsonine treated individuals is delayed by approximately one day. These sub-lethal effects observed during larval and pupal development could reflect that, as in cases of loco-poisoning in livestock, a prolonged exposure is necessary for the onset of severe symptoms; the effects on early development are subtle, and the toxicity of swainsonine only becomes apparent during late larval/pupal stages and upon emergence.

Studies of swainsonine poisoning in mammals indicate that locoism results from the progressive accumulation of partially degraded oligosaccharides causing extensive lysosomal vacuolation and tissue disruption (Broquist, 1985). This accumulation first affects the liver and kidney, causing general malaise, with eventual neuronal vacuolation causing severe neurological symptoms and death. The onset of loco-like symptoms in newly emerged bees, who display altered locomotor activity and do not survive beyond 5 days post-emergence, indicate that a prolonged exposure to swainsonine, at high concentrations, induces locoism in the bee.

Given that LAM is involved in general metabolic processes and cellular turnover, an inhibition of its activity during larval development may reduce overall larval growth, explaining the observed developmental delay during these stages. Alternatively, this delay could reflect the need for resources to be allocated away from larval growth to survive poisoning and allow detoxification processes to occur. Numerous alkaloids are toxic to the honey bee (Detzel & Wink, 1993), and in newly emerged bees exposed to the alkaloid nicotine detoxification processes are up-regulated, and this is associated with altered energy metabolism (Du Rand et al., 2015). A similar response after exposure to swainsonine could explain our findings, where fewer energetic resources are available for larval development and subsequent metamorphosis, explaining the sub-lethal effects observed in this time period.

In the honey bee LAM is very highly expressed in the digestive system during larval stages (Wedd, Kucharski & Maleszka, 2016). The significance of this finding remains unknown, but it is possible that LAM may be involved in the processing of components of the larval diet. Larval development in the bee is highly dependent on diet, and any shift in the availability of nutrients would have important implications on such development (Barchuk et al., 2007; Foret et al., 2012; Maleszka, 2008; Maleszka, 2014). The switch from royal jelly to worker jelly, for instance, has a profound effect on development, causing a larva to develop into a worker rather than a queen, and nurse bees are known to tightly regulate various components in the larval diet, such as sugar content, to guide worker development. Typically, a spike in juvenile hormone at the end of larval development initiates metamorphosis, and nurse bees increase the amount of sugar in larval food during the fifth instar to cause this spike (Barchuk et al., 2007). If swainsonine treatment causes vacuolation in the larval gut, absorption may be compromised, and the delay seen in our study may result from an alteration in the uptake of key nutrients required for metamorphosis. Indeed, swainsonine is known to induce apoptosis *in vivo*. It has been widely studied for its anti-tumour effects, and it has been shown to delay the growth of tumour cells by inducing apoptosis (Li et al., 2012; Sun et al., 2007). The administration of swainsonine via the larval diet could certainly result in the larval digestive system undergoing vacuolation and apoptosis, compromising nutrition. The abnormal locomotor activity and eventual death of newly emerged bees also suggests that such swainsonine induced apoptosis and vaculolation inevitably affects the honey bee brain.

LAM is one of numerous N-glycosylated proteins found in royal jelly (Zhang et al., 2014), many of which are high-mannose type glycoproteins (Kimura et al., 2000). The lysosomal catabolism of N-linked glycoproteins has been studied extensively and LAM is essential to such processing (Winchester, 2005). The fact that LAM itself is found in royal jelly and is highly expressed in the larval gut certainly indicates that one of the functions of LAM relates to nutrition, and interestingly, suggests it may help digest royal jelly components. Since queen bee development is entirely dependent on larvae being reared on a diet of royal jelly (Barchuk et al., 2007; Maleszka, 2008; Weaver, 1966), an interesting extension of our study would be to investigate the effect of swainsonine on queen development. Developing queens could be more sensitive to the malabsorption of larval diet and/or royal jelly glycoproteins, and so whilst worker development is only marginally delayed in response to swainsonine treatment, queen development might be more profoundly affected.

The extent to which this alkaloid affects global metabolism can be gleaned from our initial mass spectrometry analyses of swainsonine treated larvae. Forty six metabolites identified by this approach are indicative of several pathways involved in the metabolism of amino acids, polyamines, saccharides, hydroxyl acids and polyols. All detected changes are consistent with reduced metabolic output, increased apoptosis and epigenomic impacts on global gene regulation, which have been either observed or predicted for situations associated with LAM deficiencies (Borgwardt, Lund & Dali, 2014; Wedd, Kucharski & Maleszka, 2016). Whether swainsonine directly modifies certain biochemical pathways or these changes merely reflect a decline in metabolic demand associated with restricted growth remains to be established. It is clear however that LAM deficiency leads to a major disturbance in the entire metabolic network.

### Environmental implications of our findings

Several known pesticides, including the well-known neonicotinoids, are known to affect honey bee health (Guez, Belzunces & Maleszka, 2003; Hurst, Stevenson & Wright, 2014), and have been widely researched in the context of recent honey bee losses. Such pesticides have been reported to delay larval development and reduce adult longevity (Wu, Anelli & Sheppard, 2011), cause apoptosis in honey bee tissues and in the case of imidacloprid, a commonly used neonicotinoid insecticide, have been shown to induce neuronal apoptosis (Wu et al., 2015). Whilst the doses of swainsonine used in our study are unlikely to reflect the environmental exposures of honey bees foraging on the locoweed they highlight the importance of investigating locoism in the bee further.

If swainsonine exposure, at lower environmentally meaningful doses, causes similar effects on larval growth and adult longevity this has the potential to negatively impact on colony health. Since swainsonine has been found in both pollen and nectar (Burrows & Tyrl, 2013; James et al., 1978), foraging bees that are sensitive to this alkaloid could display altered behaviours, such as diminished learning and memory, and any losses of adults or shifts in larval development or nutrition would be detrimental. Undesirable developmental delays can increase sensitivity to pesticides or Varroa mites (Wu, Anelli & Sheppard, 2011), and cause changes in the division of labour within a hive, having long term effects on hive productivity and fitness (Goulson et al., 2015). Swainsonine thus presents as a potential environmental stressor for the honey bee, and should be investigated accordingly.

### Insights into AmLAM epiallelic variation

Our study has shown that the honey bee displays a similar level and mode of toxicity to swainsonine as in other organisms. These findings suggest that LAM function in the honey bee is conserved and therefore plays a similar role in glycoprotein catabolism and metabolism as has been shown in other species. The inhibition of LAM activity in developing larvae slows growth, and at this stage it is unclear whether this effect results from swainsonine induced apoptosis and general toxicity, or the inhibition of LAM activity itself. Whilst this lack of clarity makes it difficult to assign function to the differentially methylated AmLAM epialleles it does indicate that these epialleles, which cause differential LAM expression in larval stages, may cause subtle differences in larval metabolism, nutrition and the rate of development.

Phenotypic variation between individuals within a honey bee colony is known to increase the stability and overall health of a colony (Jones et al., 2004; Maleszka, 2016). A genetically diverse population promotes flexibility, and hives with higher variability have been shown to respond more adequately to external and internal cues, for instance, hives founded by multiply-mated queens display better thermoregulation and foraging outcomes that hives with progenies of single-mated queens (Jones et al., 2004). Additionally, nurse bees use their bodies to warm up brood cells to accelerate their development (Kleinhenz et al., 2003) to facilitate shifts in the timing of task specialisation and adult behaviour (Becher, Scharpenberg & Moritz, 2009). If the AmLAM epialleles modulate metabolic rate during development then their presence in the honey bee population could generate such phenotypic variation and timing of task specialisation that might be advantageous in particular contexts.

##  Supplemental Information

10.7717/peerj.3109/supp-1 Figure S1 K_*i*_ estimates of swainsonine Mannosidase activity was determined over time and initial velocity calculated in the presence of varying concentrations of swainsonine (0–8,000 nM), and the inhibition constant, K_*i*_, determined (GraphPad Prism). A) Preliminary estimates with swainsonine concentrations ranging from 40-8000 nM indicated a K_*i*_ of 107.6 ±20.6 nM (Km = 1,290 ± 232). The K_*i*_, of swainsonine was then determined with 10 concentrations of swainsonine (5–8,000 nM) using high (B) and medium (C) concentrations of enzyme B) K_*i*_ = 152.3 ±17.49 nM (Km = 1,519 ± 124) C) K_*i*_ = 223.6 ±24.41 nM (Km = 1,768 ±154) (range, 95% CI, from 117.3–278.4 nM).

10.7717/peerj.3109/supp-2 Figure S2 Effect of preincubation on swainsonine inhibition Mannosidase activity of *A. mellifera* extracts was determined in the absence swainsonine (100% activity) and in the presence of swainsonine with, or without precinubation. (A) Mannosidase activity was determined at swainsonine concentrations 10 × K _*i*_ (∼1,600 nM) and 0.3 × K _*i*_ (∼48 nM) without preincubation and with a 30 minute preincubation of enzyme with a subsequent 30× dilution into substrate. (B) Mannosidase activity was determined at swainsonine concentrations 5 × K_*i*_ (∼800 nM) and near to the K_*i*_ (160 nM) without preincubation and with a 30 min preincubation, with a subsequent 5× dilution into substrate.

10.7717/peerj.3109/supp-3 Figure S3 Principal component analysis of the GC/MS data Principal component analysis of the GC/MS data for the swainsonine treated and control samples. Key: C, untreated control sample; T, swainsonine treated samples; The first digit in each name represents the biological replicate number (e.g., C1, C2 and C3), while the second digit after the decimal space indicates the technical replicate number (e.g., C1.1, C1.2 and C1.3).

10.7717/peerj.3109/supp-4 Data S1 Raw data

10.7717/peerj.3109/supp-5 Table S1

## Additional Information and Declarations

### Data Availability

The following information was supplied regarding data availability: The raw data has been supplied as a Supplementary File.
