# Supplementary figures and images for: Developmental and loco-like effects of a swainsonine-induced inhibition of α-mannosidase in the honey bee, Apis mellifera
Source: PeerJ. 2017 Mar 16;5:e3109. doi: 10.7717/peerj.3109 (PMC5357340; doi:10.7717/peerj.3109)

**A**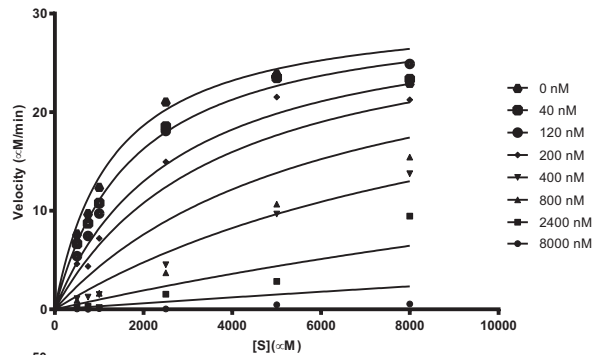**B**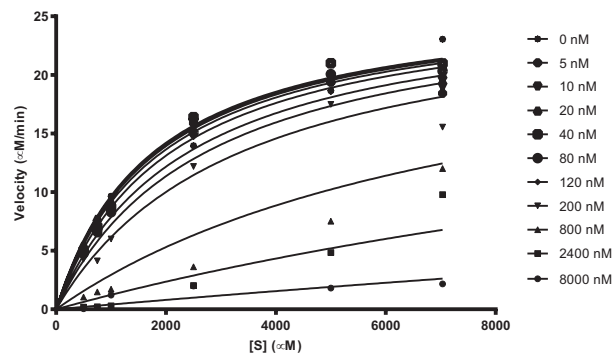**C**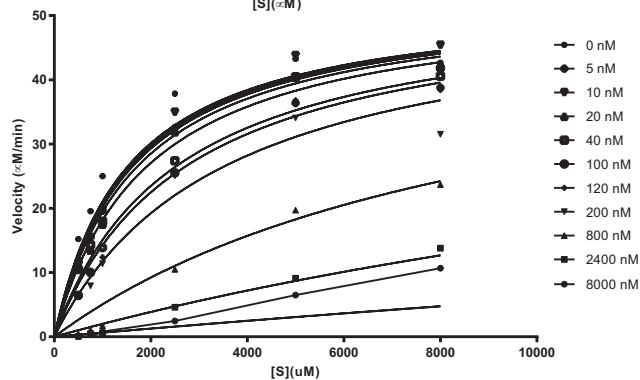

Supplement: Figure S1 — Mannosidase activity was determined over time and initial velocity calculated in the presence of varying concentrations of swainsonine (0–8,000 nM), and the inhibition constant, Ki, determined (GraphPad Prism). A) Preliminary estimates with swainsonine concentrations ranging from 40-8000 nM indicated a Ki of 107.6 ±20.6 nM (Km = 1,290 ± 232). The Ki, of swainsonine was then determined with 10 concentrations of swainsonine (5–8,000 nM) using high (B) and medium (C) concentrations of enzyme B) Ki = 152.3 ±17.49 nM (Km = 1,519 ± 124) C) Ki = 223.6 ±24.41 nM (Km = 1,768 ±154) (range, 95% CI, from 117.3–278.4 nM). [file peerj-05-3109-s001.pdf]

# Effect of preincubation on inhibition

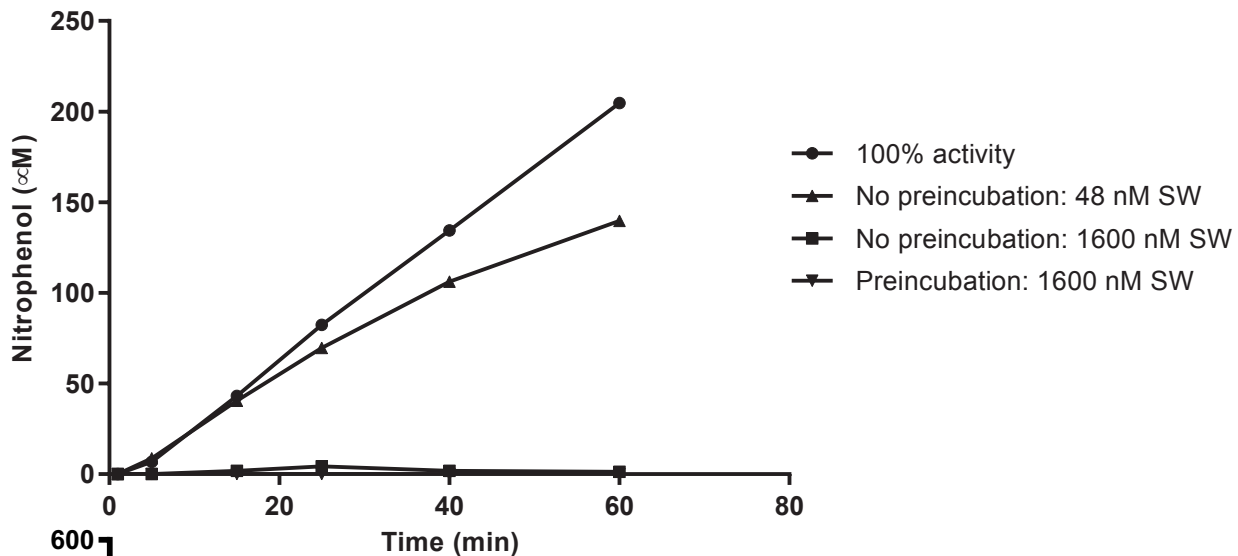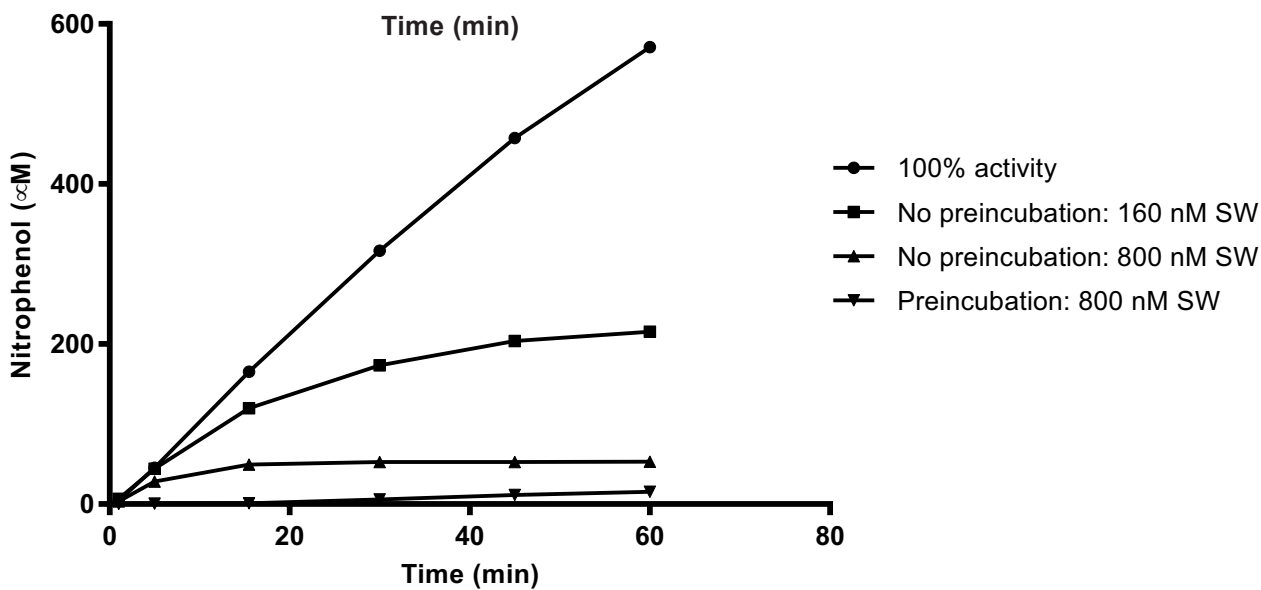

Supplement: Figure S2 — Mannosidase activity of A. mellifera extracts was determined in the absence swainsonine (100% activity) and in the presence of swainsonine with, or without precinubation. (A) Mannosidase activity was determined at swainsonine concentrations 10 × K i (∼1,600 nM) and 0.3 × K i (∼48 nM) without preincubation and with a 30 minute preincubation of enzyme with a subsequent 30× dilution into substrate. (B) Mannosidase activity was determined at swainsonine concentrations 5 × Ki (∼800 nM) and near to the Ki (160 nM) without preincubation and with a 30 min preincubation, with a subsequent 5× dilution into substrate. [file peerj-05-3109-s002.pdf]

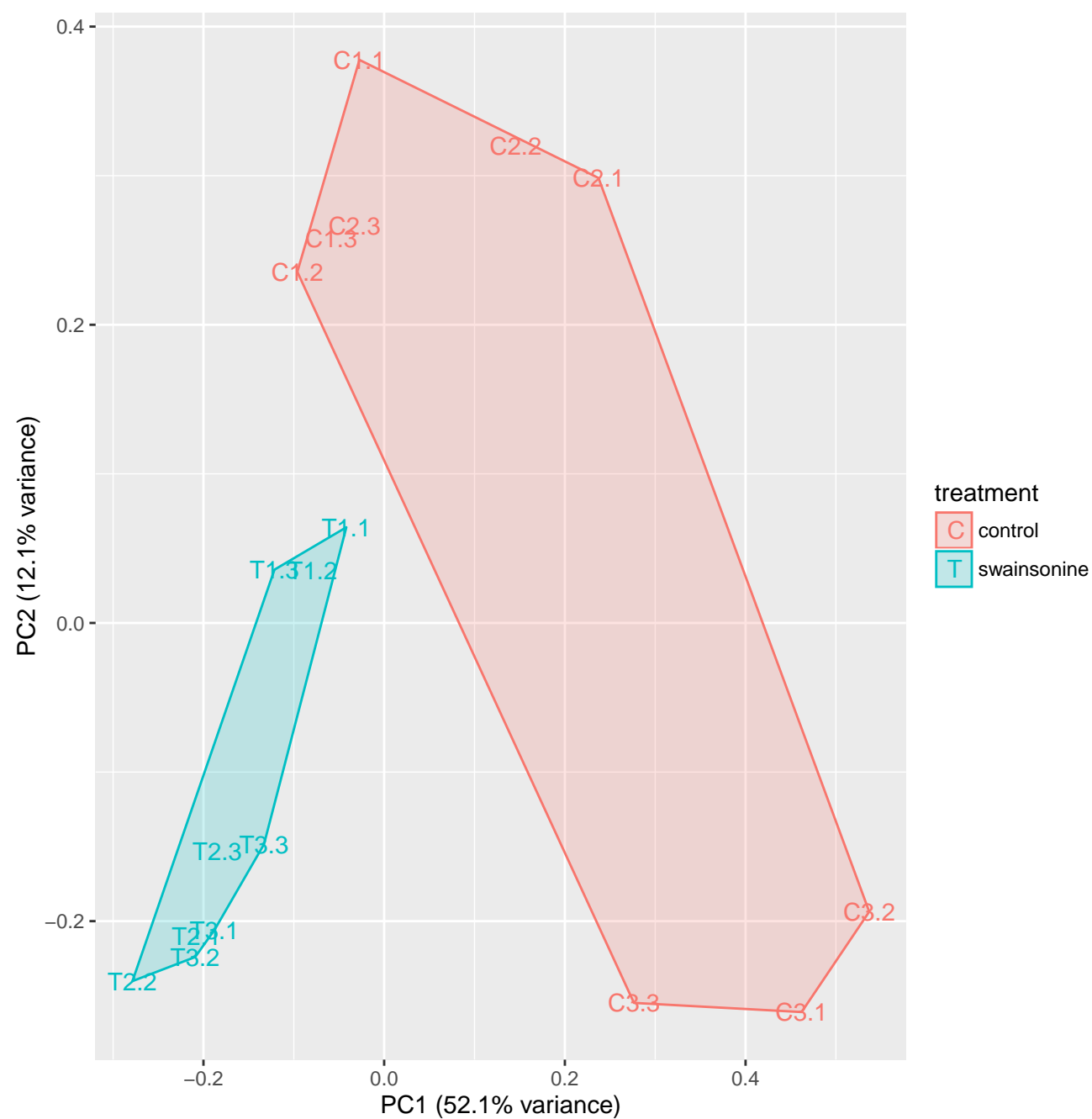

Supplement: Figure S3 — Principal component analysis of the GC/MS data for the swainsonine treated and control samples. Key: C, untreated control sample; T, swainsonine treated samples; The first digit in each name represents the biological replicate number (e.g., C1, C2 and C3), while the second digit after the decimal space indicates the technical replicate number (e.g., C1.1, C1.2 and C1.3). [file peerj-05-3109-s003.pdf]
