# Supplementary material for: Developmental and loco-like effects of a swainsonine-induced inhibition of α-mannosidase in the honey bee, Apis mellifera
Source: PeerJ. 2017 Mar 16;5:e3109. doi: 10.7717/peerj.3109 (PMC5357340; doi:10.7717/peerj.3109)
Supplement: Table S1 [file peerj-05-3109-s005.pdf]

Table S1. Full list of metabolites identified by GC/MS analyses

Analyte Class	Analyte	Analyte Signal Annotation	Average Retention Time (min)	Average Retention Index (Kovats)	Quantifier m/z	Signal Intensity Ratio	p-Value
Alcohols and Polyols	Arabitol	Arabitol (5TMS)_ID_RI1707.57_MZ129	10.595	1707.7	129	0.44	0.0380
Alcohols and Polyols	Erythritol	Erythritol (4TMS)_ID55_RI1490_MZ307	8.935	1488.9	307	0.50	0.0125
Alcohols and Polyols	Glycerol	Glycerol (3TMS)_ID210_RI1263.7_MZ132	6.962	1263.7	132	0.21	0.0225
Alcohols and Polyols	Glycerol-3-phosphate	Glycerol-3-phosphate (4TMS)_ID_RI1750.51_MZ357	10.928	1752.1	357	0.47	0.0030
Alcohols and Polyols	similar to Inositol	similar to Inositol (6TMS)_ID_RI2010.27_MZ191	12.753	2012.2	191	0.47	0.0343
Alcohols and Polyols	Sorbitol	Sorbitol (6TMS)_ID133_RI1918.6_MZ319	12.145	1916.5	319	0.42	0.0480
Alcohols and Polyols	Threitol	Threitol (4TMS)_ID_RI1485.23_MZ103	8.906	1485.6	103	0.37	0.0269
Amino Acids	2-Piperidinecarboxylic acid	2-Piperidinecarboxylic acid (1TMS)_ID244_RI1275.5_MZ186	7.069	1275.8	186	7.89	0.0478
Amino Acids	Aspartic acid	Aspartic acid (2TMS)_ID_RI1422.05_MZ160	8.346	1421.7	160	0.50	0.0094
Amino Acids	Beta-Alanine	Alanine, beta- (3TMS)_ID_RI1423.86_MZ100	8.366	1423.9	100	0.70	0.0291
Amino Acids	Cycloleucine	Leucine, cyclo- (2TMS)_ID_RI1366.92_MZ156	7.860	1366.1	156	7.43	0.0327
Amino Acids	Cysteine	L-Cysteine_ID64_RI1549.8_MZ220	9.410	1550.2	220	5.95	0.0313
Amino Acids	gamma-Aminobutyric acid	Butanoic acid, 4-amino- (3TMS)_ID_RI1526.01_MZ174	9.219	1524.9	174	0.57	0.0456
Amino Acids	Glutamine	L-Glutamine (3TMS)_ID110_RI1773.4_MZ156	11.077	1771.9	156	4.76	0.0121
Amino Acids	Histidine	Histidine (3TMS)_ID256_RI1915.4_MZ254	12.135	1914.9	254	3.52	0.0334
Amino Acids	Hydroxyproline	Proline, 4-hydroxy-, trans- (3TMS)_ID_RI1517.3_MZ230	9.156	1516.4	230	3.44	0.0455
Amino Acids	Ornithine	Ornithine (4TMS)_ID117_RI1812.2_MZ174	11.372	1811.1	174	4.11	0.0154
Amino Acids	Proline	L-Proline (2TMS)_ID22_RI1297_MZ142	7.269	1298.7	142	3.92	0.0241
Amino Acids	Serine	L-Serine (3TMS)_ID31_RI1350.6_MZ218	7.733	1351.6	218	3.17	0.0396
Amino Acids	Tyrosine	Tyrosine (3TMS)_ID_RI1934.12_MZ218	12.253	1933.4	218	0.17	0.0203
hydroxy acids	Myrmicacin	Decanoic acid, 3-hydroxy- (2TMS)_ID_RI1648.1_MZ233	10.153	1649.1	233	0.27	0.0032
hydroxy acids	Sebacic acid	Sebacic acid (2TMS)_ID_RI1885.64_MZ331	11.958	1888.9	331	0.21	0.0421
Polyamines	Cadaverine	Cadaverine (4TMS)_ID_RI1834.54_MZ174	11.552	1835.0	174	3.24	0.0447
Polyamines	Putrescine	Putrescine (4TMS)_ID105_RI1739.2_MZ214	10.836	1739.9	214	2.95	0.0258
Polyamines	Spermidine	Spermidine (5TMS)_ID163_RI2248_MZ156	14.198	2248.1	156	3.06	0.0480
Saccharide	Allose	Allose (1MEOX) (5TMS) BP_ID_RI1885.22_MZ129	11.926	1884.8	129	0.14	0.0233
Saccharide	Altrose	Altrose (1MEOX) (5TMS) MP_ID_RI1880.27_MZ319	11.883	1879.0	319	0.49	0.0020
Saccharide	Arabinose	Arabinose (1MEOX) (4TMS) MP_ID_RI1650.62_MZ103	10.167	1650.8	103	0.27	0.0036
Saccharide	Fructose	Fructose (1MEOX) (5TMS) BP_ID_RI1863.13_MZ103	11.764	1863.1	103	0.60	0.0347
Saccharide	Galactose	Galactose (1MEOX) (5TMS) MP_ID_RI1876.07_MZ319	11.884	1879.2	319	0.52	0.0015
Saccharide	Glucose	Glucopyranose (5TMS)_ID_RI1958.51_MZ191	12.353	1955.3	191	0.32	0.0095
Saccharide	Glucose	Glucose (1MEOX) (5TMS) MP_ID_RI1880.5_MZ319	11.889	1879.8	319	0.61	0.0250
Saccharide	Idose	Idose (1MEOX) (5TMS) BP_ID_RI1897.25_MZ205	12.031	1898.7	205	0.50	0.0120
Saccharide	Maltose	alpha-D-Galactopyranosyl-(1,4)-D-galactopyranoside (1MEOX) (8TMS) BP_ID_RI2782.27_MZ204	17.030	2783.2	204	0.50	0.0346
Saccharide	Mannose	Mannose (1MEOX) (5TMS) EZ Peak 1 (Major)_ID124_RI1866.1_MZ319	11.790	1866.7	319	0.33	0.0334
Saccharide	Psicose	Psicose (1MEOX) (5TMS) MP_ID_RI1849.82_MZ217	11.693	1853.8	217	0.41	0.0013
Saccharide	Ribose	Ribose (1MEOX) (4TMS) MP_ID_RI1665.99_MZ217	10.280	1666.0	217	0.50	0.0231
Saccharide	Sorbose	Sorbose (1MEOX) (5TMS) MP_ID_RI1858.01_MZ307	11.683	1857.7	307	0.46	0.0225
Saccharide	Sucrose	Sucrose (8TMS)_ID_RI2622.87_MZ129	16.163	2619.4	129	0.18	0.0472
Saccharide	Tagatose	Tagatose (1MEOX) (5TMS) MP_ID_RI1850.86_MZ217	11.694	1853.9	217	0.43	0.0016
Saccharide acid	Arabinonic acid	Arabinonic acid (5TMS)_ID_RI1764.84_MZ292	11.049	1768.1	292	0.61	0.0206
Saccharide acid	Galactonic acid	Galactonic acid (6TMS)_ID_RI1980.32_MZ205	12.572	1983.8	205	0.29	0.0012
Saccharide acid	Gluconic acid	Gluconic acid (6TMS)_ID_RI1984.83_MZ333	12.575	1984.2	333	0.27	0.0016
Saccharide acid	Glyceric acid	Glyceric acid (3TMS)_ID_RI1319.94_MZ292	7.475	1322.2	292	0.24	0.0017
Lactone	Delta-Gluconolactone	Gluconic acid-1,5-lactone (4TMS)_ID_RI1871.9_MZ220	11.834	1872.5	220	0.27	0.0010
